# Editorial: Rehabilitation, communication disorders and person-family-centered care

**DOI:** 10.3389/fresc.2024.1458662

**Published:** 2024-08-12

**Authors:** Faheema Mahomed-Asmail, Marien Alet Graham, Rachel Glade, Nannette Nicholson

**Affiliations:** ^1^ Department of Speech-Language Pathology and Audiology, University of Pretoria, Pretoria, South Africa; ^2^ Department of Early Childhood Education, University of Pretoria, Pretoria, South Africa; ^3^ Department of Communication Disorders and Occupational Therapy, University of Arkansas, Fayetteville, AR, United States; ^4^ Department of Clinical Research, NorthStar Audiology, Colorado Springs, CO, United States

**Keywords:** person-centred, family-centred, audiology, speech pathology, communication science disorders (CSD), new trends, teaching

**Editorial on the Research Topic**
Rehabilitation, communication disorders and person-family-centered care

Patient-centered care (PCC) was introduced in the psychological counseling literature nearly six decades ago and has created a change in the approach followed in healthcare, from a predominantly medical model to a biopsychosocial model of care (1, 2). Growing evidence suggests that implementing PCC may address the holistic needs of the person, eliciting a greater adherence to treatment, resulting in overall increased satisfaction (3–5). Moreover, participation and involvement of persons in their healthcare, as proposed by person-centeredness, are in line with ethical practice principles of autonomy, encouraging shared power and responsibility between patients and healthcare providers in decision-making (6, 7). PCC may also encourage self-management, which can have a positive influence on the persons experiences with the health condition (8).

Family-centered care (FCC), an extension of PCC, moves beyond the person with the disability/difficulty. The implementation of person- and family-centred care (PFCC) in healthcare is a multifaceted process that encompasses various promoting factors and obstacles (Sun et al.). The provision of PFCC is grounded on empowering persons and families to be equal partners and collaborators in their healthcare, where their preferences, needs, beliefs and culture are upheld (9). Facilitators toward PFCC include open communication, team-based care, dedicated disease-specific education (10), policies and procedures and an organizational culture that provides support and training in person-centered care (11).

While PFCC is widely advocated in the management of chronic health conditions due to its positive effect on health outcomes, its implementation in many healthcare sectors, such as communication sciences and disorders (CSD), is far from complete. The goal of this special edition was to provide a better understanding of PFCC particularly related to communication sciences disorders. The special edition was initiated in December 2022 and opened for submission from April to July 2023. A total of six articles were accepted for publication. The papers in this edition are broad in their scope, ranging from providing PCC to individuals with speech disorders to determining client and audiologist attitudes toward PCC.

The special issue begins with an exploration into the preferences and predictors toward PCC in speech-language pathology and audiology (SLP/A) clinicians in South Africa (Mahomed-Asmail et al.) using a mixed-methods approach. An overall high preference toward PCC was found with predictors toward PCC including age, home language, employment sector, and personality trait of openness. Facilitators and barriers to PCC were mentioned as areas for further investigation to improve implementation of PCC.

Barriers and facilitators to FCC, specifically in individuals with Parkinson's disease (PD), were investigated in the scoping review conducted by Sun et al. Thirty-five studies were included with the following barriers and facilitators identified: physiological factors, environmental factors, culturally based conflicts, living arrangements, education or skills training, group experiences, and individual and family consultations. It was recommended that it is necessary to clarify the connotation of FCC in PD and to also attach importance to the role healthcare providers play in delivering ongoing care.

When working with individuals with aphasia, Hinckley and Jayes mention in their narrative review article that shared decision making (SDM) is vital in rehabilitation. Their review discusses tools and strategies to support SDM, which are also applicable to various other healthcare providers. The case scenarios illustrate best practices, emphasizing the role of these tools in facilitating effective SDM in rehabilitation. Furthermore, it was mentioned that in order to help future clients and practitioners more readily engage in SDM, students in health and social services should be trained in supported communication and SDM so that they better provide PCC.

In audiology, where decisions regarding hearing healthcare have a significant impact on individuals' quality of life, implementing PCC and, more specifically, SDM is crucial as it places the client at the center of their care (12). The article by Hussain et al. used a qualitative approach to investigate audiology students' perspectives regarding the value of SDM. Findings show that audiology students view SDM as essential to their future roles. Through focus groups, students identified resources, decision aids, and the Ida Institute as pivotal to understanding SDM. It was recommended that SDM should be emphasized in audiology training to enhance clinical practices, with future research needed on the clients' perspectives in training programs.

Notably, a critical starting point to the successful delivery of PFCC is to understand who the person receiving care is (13). The study by Hlayisi and Sekoto explored identity construction among deaf adolescents and young adults (AYA) through a qualitative interpretive phenomenological approach. Findings highlight that identity construction occurs concurrently at several levels. At the personal level, AYA create self-conceived ideals of who they are. At relational level, identity is fostered through person-to-person and person-to-group interactions. At societal level, AYA navigate inherent challenges with hearing loss and their positionality as deaf individuals.

It is important to note that when applied to audiological care, PFCC should not fixate on the hearing loss or communication disorder alone but should contextualize audiological care with the broader psychosocial aspects of the person's livelihood in mind (Hussain et al.) In the study by Warren and Barron, audiologists working with cochlear implant users recognized the benefits of psychosocial interventions but faced barriers in addressing these needs, such as lack of time and comfort in counseling. Despite acknowledging the importance of psychosocial care, the majority (93%) of clinicians reported that they never screen for psychosocial symptoms, with referrals occurring less than half the time or never. This study found that strategies to enhance psychosocial care recognition and interprofessional practice are needed to improve PCC in hearing healthcare.

## Conclusion

The special edition highlights facilitators and barriers to PFCC, emphasizing the need for open communication, team-based care, and supportive organizational cultures. Studies also underscore the importance of SDM and psychosocial interventions in enhancing patient-centered care, particularly in audiology and speech-language pathology. To advance PFCC, future efforts should focus on training healthcare professionals in SDM and supported communication, as well as addressing barriers to comprehensive psychosocial care.
